# Supplementary material for: Serum Metabolomic Profiling in Aging Mice Using Liquid Chromatography—Mass Spectrometry
Source: Biomolecules. 2022 Oct 29;12(11):1594. doi: 10.3390/biom12111594 (PMC9687663; doi:10.3390/biom12111594)
Supplement: Supplementary file 1 [file biomolecules-12-01594-s001.zip › biomolecules-1943351-supplementary.pdf]

Supplementary materials for

Plasma metabolomic profiling in ageing mouse using liquid chromatography-mass spectrometry

This file includes:

Supplementary Figure S1

Supplementary Tables S1–S3

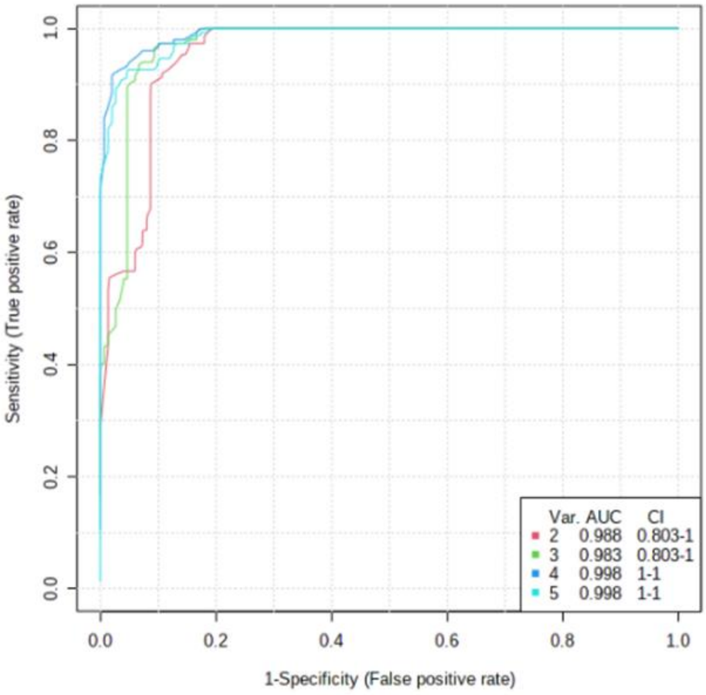

Supplementary Figure S1. ROC curve of the candidate biomarkers

Supplementary Table S1. Composition of normal chow diet. （fish meal, soy bean, wheat, corn, bran, beer yeast, soybean oil, vitamin, mineral mix, amino acid; Isoflavone content < 20mg/kg）

Composition of normal chow diet	
Diet	Normal diet
Energy composition (%)	
Carbohydrate	74.90%
Protein	20.30%
Fat	4.80%
Ingredients(g/Kg)	
Casein	200
L-Cystine	3
Com starch	397
Maltodextrin	132
Sucrose	100
Cellulose	50
Soybean Oil	70
t-Butylhydroquinone	0.014
Lard oil	0
Mineral Mix	35
Di Calcium Phosphate	0
Calcium Carbonate	0
Potassium Bitartrate	0
Vitamin Mix	10
Choline Bitartrate	2.5
FD&C Red Dye	0

Supplementary Table S2. Pathway analysis of significant altered metabolites (pos: red color; neg: blue color)

POS.Pathway	Count	Count.All	Pvalue	Pathway.ID	KEGG.Names	KEGG.IDs
Biosynthesis of amino acids	6	128	0.000255588	map01230	L-homoserine;L-methionine;Citric acid;Alpha-ketoglutaric acid;(2s,4s)-4-hydroxy-2,3,4,5-tetrahydrodipicolinic acid;Glycine	C00263+C00073+C00158+C00026+C20258+C00037
Caffeine metabolism	3	22	0.000443235	map00232	Theobromine;Theophylline;Caffeine	C07480+C07130+C07481
Cysteine and methionine metabolism	4	61	0.000819138	map00270	L-homoserine;L-methionine;Ophthalmic acid;Formyl-l-methionyl peptide	C00263+C00073+C21016+C03145
Linoleic acid metabolism	3	28	0.00091495	map00591	(+/-)12(13)-dihome;9-oxo-10(e),12(e)-octadecadienoic acid;Gamma-linolenic acid	C14829+C14766+C06426
Metabolic pathways	22	1706	0.002848024	map01100	4-guanidinobutyric acid;L-homoserine;L-methionine;Citric acid;Alpha-ketoglutaric acid;Ophthalmic acid;(2s,4s)-4-hydroxy-2,3,4,5-tetrahydrodipicolinic acid;D-(-)-glutamine;Bilirubin;4-acetamidobutanoate;Theobromine;Pantothenic acid;Theophylline;4-(2-aminophenyl)-2,4-dioxobutanoic acid;Caffeine;Caprolactam;Glycine;Methyl (2r,3s)-3-hydroxy-8-methyl-8-azabicyclo[3.2.1]octane-2-carboxylate;3-tropanol;Jasmonic acid;12-oxo phytodienoic acid;Gamma-linolenic acid	C01035+C00263+C00073+C00158+C00026+C21016+C20258+C00819+C00486+C02946+C07480+C00864+C07130+C01252+C07481+C06593+C00037+C12448+C00729+C08491+C01226+C06426
D-Glutamine and D-glutamate metabolism	2	12	0.002954462	map00471	Alpha-ketoglutaric acid;D-(-)-glutamine	C00026+C00819
alpha-Linolenic acid metabolism	3	44	0.003414796	map00592	Jasmonic acid;12-oxo phytodienoic acid;Methyl jasmonate	C08491+C01226+C11512
Lysine degradation	3	54	0.006085045	map00310	Alpha-ketoglutaric acid;6-acetamido-2-oxohexanoic acid;Glycine	C00026+C05548+C00037

Citrate cycle (TCA cycle)	2	20	0.008205431	map00020	Citric acid;Alpha-ketoglutaric acid	C00158+C00026
Glyoxylate and dicarboxylate metabolism	3	62	0.008917245	map00630	Citric acid;Alpha-ketoglutaric acid;Glycine	C00158+C00026+C00037
Glucagon signaling pathway	2	26	0.01366424	map04922	Citric acid;Alpha-ketoglutaric acid	C00158+C00026
2-Oxocarboxylic acid metabolism	4	134	0.0137468	map01210	L-methionine;Citric acid;Alpha-ketoglutaric acid;1778635	C00073+C00158+C00026+C16589
Alanine, aspartate and glutamate metabolism	2	28	0.01575144	map00250	Citric acid;Alpha-ketoglutaric acid	C00158+C00026
Mineral absorption	2	29	0.016843	map04978	L-methionine;Glycine	C00073+C00037
Protein digestion and absorption	2	47	0.04140663	map04974	L-methionine;Glycine	C00073+C00037
Histidine metabolism	2	47	0.04140663	map00340	1-methylhistamine;Alpha-ketoglutaric acid	C05127+C00026
Carbon metabolism	3	112	0.04203641	map01200	Citric acid;Alpha-ketoglutaric acid;Glycine	C00158+C00026+C00037
Fatty acid biosynthesis	2	50	0.04630623	map00061	Palmitoleic acid;Oleate	C08362+C00712
Glycine, serine and threonine metabolism	2	50	0.04630623	map00260	L-homoserine;Glycine	C00263+C00037
Aminoacyl-tRNA biosynthesis	2	52	0.04968497	map00970	L-methionine;Glycine	C00073+C00037

NEG.Pathway	Count	Count.All	Pvalue	Pathway.ID	KEGG.Names	KEGG.IDs
Fatty acid biosynthesis	3	50	0.000419063	map00061	Oleic acid;Myristic acid;Palmitoleic acid	C00712+C06424+C08362
Phenylalanine metabolism	3	60	0.000716723	map00360	Hippuric acid;2-hydroxyphenylacetic acid;3-phenyllactic acid	C01586+C05852+C05607

Cysteine and methionine metabolism	2	61	0.01372876	map00270	L-(-)-methionine;Fmet	C00073+C03145
Vascular smooth muscle contraction	1	16	0.0457461	map04270	11,12-epoxy-(5z,8z,11z)-icosatrienoic acid	C14770

Supplementary Table S3. Serum biomarkers identified in the negative and positive modes by machine learning.

Mode	Compound ID	Name	Formula	KEGG.ID	HMDB.ID	level	Super.class	Pathway
_	10.311_282.256	Oleic acid	C18 H34 O2	C00712	HMDB0000207	Level2	Lipids and lipid-like molecules	map00061 Fatty acid biosynthesis; map01040 Biosynthesis of unsaturated fatty acids;
_	3.533_179.0616	Cyclamic acid	C6 H13 N O3 S	C02824	HMDB0031340	Level4	Organic acids and derivatives	NA
_	0.677_90.0317	Dl-lactic acid	C3 H6 O3	C01432	HMDB0144295	Level2	Organic acids and derivatives	NA
_	9.049_320.2352	11,12-epoxy-(5z,8z,11z)-icosatrienoic acid	C20 H32 O3	C14770	NA	Level2	FA Fatty acyls	map00590 Arachidonic acid metabolism; map01100 Metabolic pathways; map04270 Vascular smooth muscle contraction; map04726 Serotonergic synapse; map04913 Ovarian steroidogenesis;
_	9.673_613.3386	NA	C31 H52 N O9 P	NA	NA	Level5	NA	NA
_	9.691_589.3388	NA	C29 H52 N O9 P	NA	NA	Level5	NA	NA
_	9.853_254.2247	Palmitoleic acid	C16 H30 O2	C08362	HMDB0003229	Level2	Lipids and lipid-like molecules	map00061 Fatty acid biosynthesis;
_	9.967_304.2404	Arachidonic acid	C20 H32 O2	C00219	HMDB0001043	Level2	Lipids and lipid-like molecules	map00590 Arachidonic acid metabolism; map00591 Linoleic acid metabolism; map01040 Biosynthesis of unsaturated fatty acids; map01100 Metabolic pathways; map04216 Ferroptosis; map04217 Necroptosis; map04270 Vascular smooth muscle contraction; map04611 Platelet activation; map04664 Fc epsilon RI signaling pathway; map04666 Fc gamma R-mediated phagocytosis; map04723

									Retrograde endocannabinoid signaling; map04726 Serotonergic synapse; map04730 Long-term depression; map04750 Inflammatory mediator regulation of TRP channels; map04912 GnRH signaling pathway; map04913 Ovarian steroidogenesis; map04921 Oxytocin signaling pathway; map04923 Regulation of lipolysis in adipocytes; map04925 Aldosterone synthesis and secretion; map05140 Leishmaniasis; map05146 Amoebiasis;
–	9.917_328.2405	Docosahexaenoic acid	C22 H32 O2	C06429	HMDB0002183	Level2	Lipids and lipid-like molecules	map01040 Biosynthesis of unsaturated fatty acids;
–	10.005_280.2403	Linoleic acid	C18 H32 O2	C01595	HMDB0000673	Level2	Lipids and lipid-like molecules	map00591 Linoleic acid metabolism; map01040 Biosynthesis of unsaturated fatty acids; map01100 Metabolic pathways;
+	8.101_317.2347	4,17-dimethyl-4-aza-5-androsten-17-ol-3-one	C20 H31 N O2	317.2347	8.101	Level4	C15175	S205466
+	7.518_273.2669	Hexadecasphinganine	C16 H35 N O2	273.2669	7.518	Level4	C13915	S571105
+	9.997_358.3083	1-stearoylglycerol	C21 H42 O4	358.3083	9.997	Level2	D01947	MReference-939
+	0.759_203.116	O-acetylcarnitine	C9 H17 N O4	C02571	NA	Level1	NA	map04931 Insulin resistance;
+	1.305_164.0477	2-hydroxycinnamic acid	C9 H8 O3	C01772	HMDB0002641	Level2	Phenylpropanoids and polyketides	map00360 Phenylalanine metabolism; map01100 Metabolic pathways;
+	9.976_380.2901	NA	C19 H36 N6 O2	NA	NA	Level5	NA	NA
+	7.539_317.2931	2-amino-1,3,4-octadecanetriol	C18 H39 N O3	C12144	HMDB0004610	Level2	Organic nitrogen compounds	map00600 Sphingolipid metabolism; map01100 Metabolic pathways;
+	9.684_328.2399	Docosahexaenoic acid	C22 H32 O2	C06429	HMDB0002183	Level2	Lipids and lipid-like molecules	map01040 Biosynthesis of unsaturated fatty acids;

+	2.711_174.1095	NA	NA	NA	NA	Level5	NA	NA
+	2.709_157.0829	[similar to: trans,trans-2,4-heptadienal]	NA	NA	NA	Level5	NA	NA
